# Human Body Odour Composites Are Not Perceived More Positively than the Individual Samples

**DOI:** 10.1177/2041669518766367

**Published:** 2018-05-07

**Authors:** Jitka Fialová, Agnieszka Sorokowska, S. Craig Roberts, Lydie Kubicová, Jan Havlíček

**Affiliations:** Faculty of Science, Charles University, Prague, Czech Republic; National Institute of Mental Health, Klecany, Czech Republic; Institute of Psychology, University of Wroclaw, Poland; Smell & Taste Clinic, Department of Otorhinolaryngology, TU Dresden, Germany; School of Natural Sciences, University of Stirling, UK; Faculty of Humanities, Charles University, Prague, Czech Republic; Faculty of Science, Charles University, Prague, Czech Republic; National Institute of Mental Health, Klecany, Czech Republic

**Keywords:** heterozygosity, averageness, odour blend, olfaction, MHC, mate preferences

## Abstract

It is well established that composite facial images are perceived as more attractive compared with individual images, suggesting a preference for heterozygosity. Similarly, there is evidence that preferences for body odours might be linked to heterozygosity. Here, we tested whether blending individual body odours into composites would follow a similar pattern as observed in the perception of faces. We collected axillary odour samples from 38 individuals, which were subsequently assessed individually and as composites of two (*N* = 19) or four (*N* = 9) body odours regarding their pleasantness, attractiveness and intensity. We found no significant differences between mean ratings of individual odour samples or composites of two or four odour samples. Our results indicate that, in contrast to faces, composite body odours are not rated as more attractive. Composite body odours retain similar hedonic perceptual qualities as individual odours, thus highlighting differences in visual and chemosensory perceptual mechanisms.

## Introduction

A robust body of evidence indicates that composite facial images are perceived as more attractive compared with the mean attractiveness rating of their constituent images. The phenomenon was first noted by Galton (1879) who used photographic superimposing techniques to combine facial images. More recent studies have improved upon these techniques, using computer graphics to generate composite facial images (Grammer & Thornhill, 1994; Langlois, Roggman, & Musselman, 1994; Rhodes, Sumich, & Byatt, 1999; Rhodes & Tremewan, 1996), and finding that composites are considered more attractive than almost all constituent faces. Furthermore, there is generally a positive association between the number of individual images constituting a composite image and its attractiveness and averageness ratings (Langlois & Roggman, 1990), although average faces may not be the most attractive (Perrett, May, & Yoshikawa, 1994).

The attractiveness of average faces may result from two different mechanisms. First, it might be a by-product of visual system processing by which average faces can be processed faster because they resemble a mental representation of a prototypical face (Enquist & Arak, 1994). Indeed, it appears that prototypes are processed faster and have a higher probability of recognition (Posner & Keele, 1968; Smith, Shoben, & Rips, 1974), and visual processing is more fluent as reflected in decreased activity in the posterior occipital cortex (Aizenstein et al., 2000). In addition, there is a positive relationship between prototypicality and preference, consistent with the observation that an increase in processing fluency also increases liking (Martindale & Moore, 1988; Whitfield & Slatter, 1979). An alternative explanation proposes that facial averageness might be a marker of heterozygosity (i.e., genetic diversity at certain loci; Lie, Rhodes, & Simmons, 2008; Thornhill & Gangestad, 1993). It has therefore been argued that attractiveness of average faces might be a perceptual adaptation to favour heterozygous individuals (Thornhill & Gangestad, 1993). Heterozygosity in some loci positively affects viability of the organism, as deleterious alleles are predominantly expressed in recessive fashion (i.e., only in homozygotes). Further, benefits of mating with heterozygous individuals may include reduced risk of disease transmission and potential for high-quality paternal care (Kirkpatrick & Ryan, 1991; Roberts et al., 2005).

Facial attractiveness and averageness is associated with heterozygosity in the genes of the major histocompatibility complex (MHC; Lie et al., 2008; Roberts et al., 2005). The MHC complex appears to be involved in mate selection processes of various vertebrate species including humans (Kamiya, O’Dwyer, Westerdahl, Senior, & Nakagawa, 2014; Winternitz, Abbate, Huchard, Havlíček, & Garamszegi, 2017). MHC genes are extremely polymorphic and code for cell-surface peptides that are responsible for recognition of foreign antigens and thereby initiating an immune response. As the MHC is expressed codominantly, heterozygous individuals are able to present a broader spectrum of peptides and thus provide resistance to a wider range of pathogens compared with MHC homozygotes (Havlíček & Roberts, 2009). Consistent with this, MHC heterozygotes have faces that appear more healthy (Roberts et al., 2005). There is also some evidence showing preferences for body odour of MHC heterozygotes: Male body odour attractiveness as rated by women was positively associated with MHC heterozygosity, but there was no similar pattern when men rated female body odour samples (Thornhill et al., 2003).

Based on this evidence, one may speculate whether composite body odours might perceptually mimic odours of heterozygote individuals, as has been observed in studies of composite faces. If so, one would predict that body odour composites—individual odours blended and presented together—would be rated more positively than the individual samples. A test of this prediction could have important consequences for the methodology of body odour studies. Numerous previous studies have presented composite odours to assessors to test potential communicatory significance of body odours. They use composite odours in an attempt to achieve a representative odour sample associated with a particular characteristic or state of interest while reducing the importance of individual variability in odour profiles. Thus, the rationale behind these studies is that composite body odours (usually created by pooling odour samples from people with a given characteristic) will contain shared qualities based on their group attributes, such as gender (Lübke et al., 2014; Mutic, Moellers, Wiesmann, & Freiherr, 2016) and sexual orientation (Martins et al., 2005). Similarly, composite body odours have been used for testing various affective states such as happiness and fear (Chen & Haviland-Jones, 2000), disgust (de Groot, Smeets, Kaldewaij, Duijndam, & Semin, 2012), sadness (Gelstein et al., 2011), anxiety (Haegler et al., 2010; Pause, Adolph, Prehn-Kristensen, & Ferstl, 2009; Pause, Lübke, Laudien, & Ferstl, 2010; Pause, Ohrt, Prehn, & Ferstl, 2004; Prehn-Kristensen et al., 2009; Zernecke et al., 2011), and stress (Dalton, Mauté, Jaén, & Wilson, 2013; Mujica-Parodi et al., 2009; Prehn, Ohrt, Sojka, Ferstl, & Pause, 2006; Radulescu & Mujica-Parodi, 2013). While the approach used in such studies is potentially very useful, it is currently unknown whether creating such composites might influence hedonic qualities.

In light of this, here we aimed to test the effects of composite odour creation on the perception of odour attractiveness, pleasantness and intensity. We created odour composites comprising two and four individual odours and tested whether these composite body odours are rated more positively compared with the distinct odours from the same individuals.

## Methods

### Raters

The individual samples were assessed by 110 raters (56 men, mean age 24.1 years, range 18–34; 54 women, mean age 23.4, range 19–35) as part of previously reported studies (Fialová & Havlíček, 2012; Fialová, Roberts, & Havlíček, 2016). The composite samples were assessed by 98 raters (50 men, mean age 23.9, range 19–33; 48 women, mean age 22.8, range 19–35). The two sets of raters were independent—no rater assessed both individual and composites. The raters were mostly Charles University students and were contacted via e-mail, posters or by oral invitation. Raters reported no respiratory or other diseases at the time of the study or any problems or medication that could influence their olfactory abilities. All women were using hormonal contraception to avoid changes in olfactory perception during the menstrual cycle (Martinec Nováková, Havlíček, & Roberts, 2014). Following the procedure used in previous studies, we assumed no systematic fluctuation in olfactory ability over time due to hormonal contraceptive use, and thus scheduling of assessments was unrestricted (e.g., Fialová et al., 2016; Kohoutová, Rubešová, & Havlíček, 2011; Sobotková, Fialová, Roberts, & Havlíček, 2017). Raters received 100 CZK (approximately US$5) as compensation for their time.

The study was conducted according to the guidelines laid down in the Declaration of Helsinki, and all procedures involving human subjects were approved by the institutional review board of Charles University, Faculty of Science. Written informed consent was obtained from all participants.

### Odour Stimuli

Twenty-six men (mean age 25.2 years; range 18–34) and 12 women (mean age 22.4; range 20–26), mostly students at Charles University (Prague, Czech Republic), participated as odour donors (sample size varies across men and women because the participants were originally recruited for the purpose of two other studies). All donors were nonsmokers and reported no dermatological or other diseases at the time of the study. No men shaved their armpits, while all women shaved their armpits; axillary shaving was kept constant within sex as it might affect perceived quality of the axillary odour (Kohoutová et al., 2011). All women used hormonal contraception to avoid possible body odour quality fluctuations across the menstrual cycle (Havlíček, Dvořáková, Bartoš, & Flegr, 2006; Kuukasjarvi et al., 2004). Male and female donors were given 400 CZK (approximately US$20) and 1,000 CZK (approximately US$40), respectively, in compensation for their time and potential inconvenience caused by the prescribed diet and according to duration of the study and its demands.

The odour donors were asked to avoid consuming smelly and spicy food, alcohol, smoking or using any cosmetics on the day before and during the sampling day (48 hr overall). They attached 100% cotton pads under each armpit using surgical tape and wore the pads for 12 h overnight (see Havlíček, Lenochová, Oberzaucher, Grammer, & Roberts, 2011). To avoid odour contamination from extrinsic ambient odours, the donors were asked to wear as the first layer of clothing a new white 100% cotton T-shirt that had been previously washed without washing powder. In the morning, they placed the pads in ziplock plastic bags and returned them to the experimenters. The samples were immediately placed in a freezer at −21℃ to prevent further microbial action and possible changes in odour quality. Freezing has been shown to have no significant effect on hedonic ratings (Lenochova, Roberts, & Havlicek, 2008). Time elapsed between removing the pads and onset of freezing was approximately 1 to 2 hr. Each donor’s conformity with the instructions was checked by a questionnaire, and no violations on the day of sampling were recorded.

To create composite body odours, we first ranked the individual samples of either sex based on their attractiveness ratings. Male and female composite odours were then created by blending two or four individual samples. We used previously unused pads that had been collected at the same time from each individual; to do this, we used the pads collected from the other armpit than the one that had been used for previous ratings of the individual samples. Each pad was cut into half and pooled with halved pads from other same-sex individuals who lay adjacent along the attractiveness continuum. In this way, we obtained 19 composites comprised of 2 individual samples and 9 composites comprised of 4 individual samples.

### Odour Rating Procedure

Ratings took place in a quiet, ventilated room. The samples from one randomly chosen armpit of each odour donor were assessed individually, by the first set of raters. The second set of raters assessed the composites, originating from the other armpit and pooled as described earlier. Both kinds of samples were presented in 250 ml opaque jars labelled with a code. Participants were asked to sniff each jar; ratings were recorded immediately after sniffing each stimulus, but the time spent sniffing was not restricted. To avoid adaptation, the samples were randomly split into subsets, and raters were given approximately 10 min break between assessing each set. All samples were thawed before the rating session and then assessed in a randomized order regarding their (a) pleasantness, (b) attractiveness and (c) intensity, each on a 7-point scale. Both ends of each scale were verbally anchored by descriptors (e.g., *very unpleasant* and *very pleasant*). If raters found any of the samples too weak to assess, they could select an option “I cannot smell the sample” instead of rating using the scales (this occurred for 4.9% of the individual samples, 3.4% of the two-composite body odour samples, and 4% of the four-composite body odour samples). Such instances were not included in analyses, and hence the sample sizes of analyses can vary.

### Statistical Analysis

Kolmogorov–Smirnov tests showed normal data distribution for all dependent variables. We computed mean values from the ratings of the individual samples that were used for creating composite body odour samples and compared them with actual ratings of composite body odour samples using paired *t* tests (e.g., for the two-odour composites, the ratings of 19 composites were compared with 19 average scores from their constituent individual odour pairs). To investigate sex differences in ratings of composite body odour samples, we used a two-way analysis of variance (ANOVA) with odour type and sex as a factor (two levels: two- and four-composite body odours and two levels: male, female, respectively). To explore whether composite body odour samples retain qualities of the individual odours, we first ranked the individual samples of either sex based on their attractiveness ratings and then split two- and four-composite body odours by the median value of the constituent samples. Subsequently, we compared the above median and below median samples by an independent samples *t* test. In what follows, mean values calculated from ratings of the individual samples and actual ratings of the composite body odour samples are referred to as calculated samples and rated samples, respectively. We also tested for possible relationships between individual samples and composite body odour samples using bivariate correlations.

## Results

### Ratings of the Individual Versus Composite Body Odours

**Figure 1.**
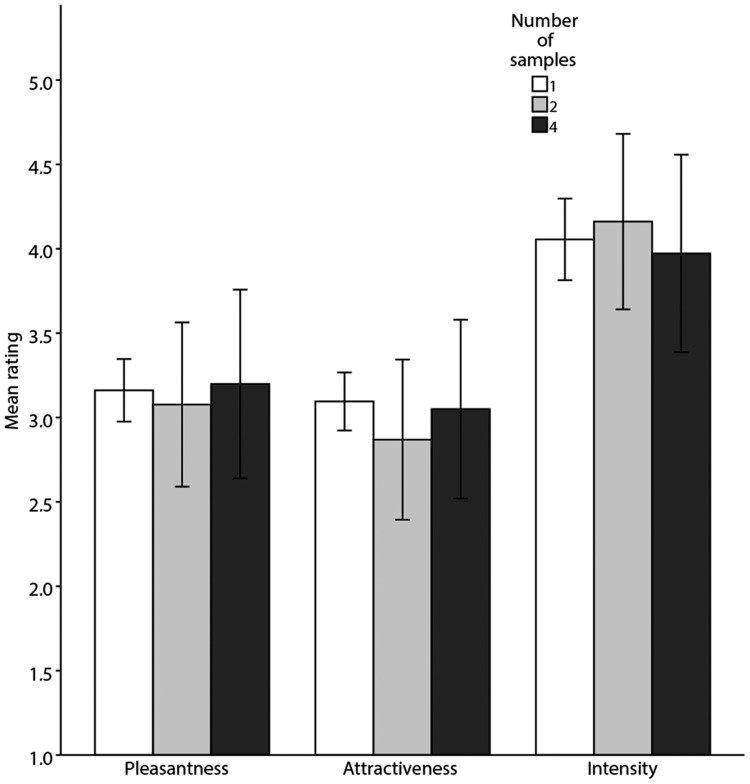
Mean ratings (± 95% CI) of calculated individual (white bars), rated two- (light grey bars) and four- (dark grey bars) composite body odour samples concerning their pleasantness, attractiveness and intensity. The individual samples were assessed by 110 raters and composite stimuli by 98 raters using 7-point scale (e.g., 1 = *very unpleasant* and 7 = *very pleasant*).

We found no significant differences between mean ratings of the individual samples and composite body odour samples (Figure 1; all *p*s > .16; for detailed results, see Tables 1 and 2). These means were calculated from the full sample of raters (i.e., both male and female raters). Subsequently, we performed a two-way ANOVA for ratings of only opposite-sex odour samples. This also did not reveal any significant differences in women’s ratings of individual, two- and four-composite male odour samples, neither for pleasantness, *F*(2, 68) = 0.008, *p* = .992; attractiveness, *F*(2, 68) = 0.159, *p* = .854 or intensity, *F*(2, 68) = 0.287, *p* = .752. Similarly, there were no significant differences in men’s ratings of individual, two- and four-composite female odour samples, for pleasantness, *F*(2, 30) = 0.605, *p* = .553; attractiveness, *F*(2, 30) = 0.726, *p* = .492 or intensity, *F*(2, 30) = 0.017, *p* = .983.

**Table 1.** Differences Between Mean Ratings (±*SD*) of Pleasantness, Attractiveness and Intensity of the Actually Rated Composite Body Odour Samples and Mean Values Calculated From Ratings of the Respective Individual Samples.

Composite	Characteristic		*M* (*SD*)	*t*	*df*	*p*	Cohen’s *d*
2	Pleasantness	Rated	3.08 (1.0)	0.56	18	.58	0.1
		Calculated	3.17 (0.73)				
2	Attractiveness	Rated	2.87 (0.98)	1.48	18	.16	0.37
		Calculated	3.13 (0.16)				
2	Intensity	Rated	4.16 (1.08)	0.63	18	.53	0.17
		Calculated	4.03 (0.19)				
4	Pleasantness	Rated	3.2 (0.73)	1.11	8	.3	0.25
		Calculated	3.38 (0.72)				
4	Attractiveness	Rated	3.05 (0.69)	2.11	8	.67	0.35
		Calculated	3.29 (0.68)				
4	Intensity	Rated	3.97 (0.76)	0.59	8	.57	0.18
		Calculated	3.81 (0.96)

**Table 2.** Comparison of Mean Ratings (± *SD*) of the Individual Samples and Two- and Four-Composite Body Odour Samples Concerning Their Pleasantness, Attractiveness and Intensity.

Characteristic	Individual sample/composite	*M* (*SD*)	Paired samples		*t*	*df*	*p*	Cohen’s *d*
Pleasantness	1	3.16 (0.81)	1	2	0.25	18	.8	0.09
	2	3.08 (1)	1	4	0.48	8	.64	0.05
	4	3.2 (0.73)	2	4	1.02	8	.34	0.14
Attractiveness	1	3.1 (0.75)	1	2	0.98	18	.34	0.26
	2	2.87 (0.98)	1	4	0.08	8	.94	0.07
	4	3.05 (0.69)	2	4	0.87	8	.41	0.21
Intensity	1	4.06 (1.06)	1	2	0.35	18	.73	0.09
	2	4.16 (1.08)	1	4	0.17	8	.87	0.1
	4	3.97 (0.76)	2	4	0.62	8	.55	0.2

**Figure 2.**
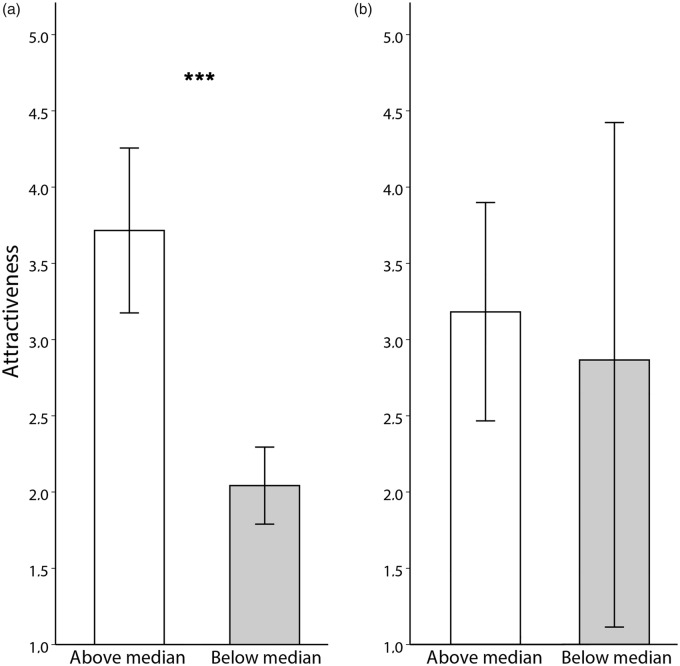
Mean attractiveness ratings (± 95% CI) of (a) two-composite body odours and (b) four-composite body odours above (white bars) and below (grey bars) the median. Asterisk indicates level of significance; ***p < .001 level.

**Figure 3.**
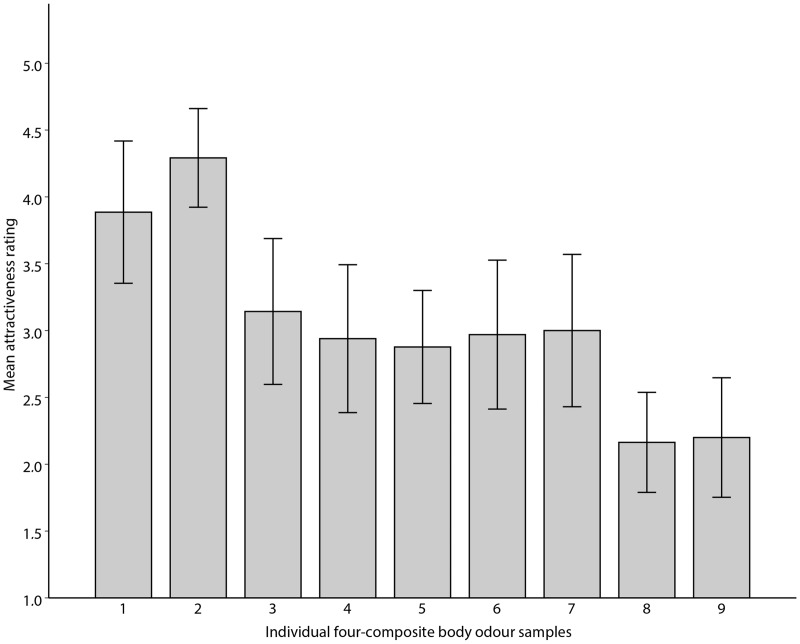
Mean attractiveness ratings (± 95% CI) of four-composite body odour samples. Numbers at individual bars indicate rank of each sample based on mean values calculated from attractiveness ratings of the respective individual odours.

To test whether composite body odours retain properties of the individual samples, we split two- and four-composite body odours by the median value of the constituent samples. We found that two-composite body odours consisting of above median samples were perceived as more pleasant, *t*(16) = 6.213, *p* < .001; attractive, *t*(16) = 6.466, *p* < .001 and intense, *t*(16) = 7.756, *p* < .001, compared with below median samples. However, differences were not significant in four-composite body odours either in pleasantness, *t*(6) = 0.437, *p* = .678; attractiveness, *t*(6) = 0.601, *p* = .570 or intensity, *t*(6) = − 0.387, *p* = .712; see Figure 2(a) and (b). For explorative purposes, we further performed a one-way ANOVA comparing individual four-composite samples and found significant differences in their attractiveness, *F*(8, 349) = 9.744, *p* < .001. A Tukey post hoc test revealed that Sample 1 was rated significantly more attractive than Sample 5 (*p* = .042) and Samples 8 and 9 (*p* < .001). Moreover, Sample 2 was significantly more attractive than all samples (*p* = .05) except Sample 1 (see Figure 3).

**Figure 4.**
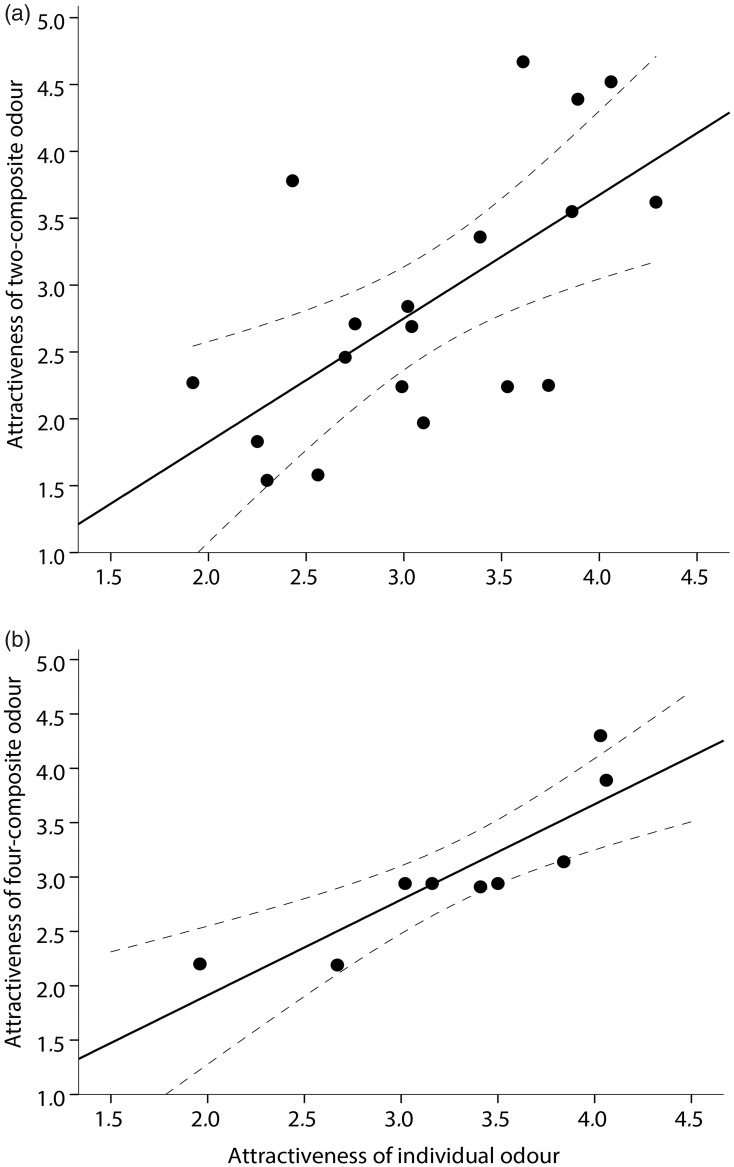
Positive correlation between attractiveness ratings of (a) two-composite body odour samples (*r* = .63) and (b) four-composite body odour samples (*r* = .87) and mean values calculated from ratings of the respective individual odours. Dashed lines indicate 95% CI.

### Sex Differences in Ratings of the Composite Body Odours

A two-way ANOVA showed that male and female ratings did not significantly differ in pleasantness, *F*(1, 52) = 1.092, *p* = .301; attractiveness, *F*(1, 52) = 1.143, *p* = .290 and intensity, *F*(1, 52) = 0.575, *p* = .452, nor was there any interaction between rater sex and number of composite body odour samples, for pleasantness, *F*(1, 52) = 0.040, *p* = .842; attractiveness, *F*(1, 52) = 0.108, *p* = .743 or intensity, *F*(1, 52) = 0.060, *p* = .807.

### Correlation Between Individual and Composite Body Odours

We found significant positive correlations between ratings of two- and four-composite body odour samples and mean values of the individual odours, for pleasantness (two-samples: *r* = .663, *p* = .002; four-samples: *r* = .783, *p* = .013), attractiveness (two-samples: *r* = .647, *p* = .003; four-samples: *r* = .873, *p* = .002) and intensity (two-samples: *r* = .603, *p* = .006; four-samples: *r* = .589, *p* = .095; see Figure 4(a) and (b), Tables 3 and 4).

**Table 3.** Correlation Between Ratings of Two-Composite Body Odour Samples (Rated—R) and Individual Odours (Calculated—C).

	Pleasantness R	Attractiveness R	Intensity R	Pleasantness C	Attractiveness C	Intensity C
Pleasantness R		.998***	−.862***	.663**	.652**	−.515*
Attractiveness R			−.856***	.647**	.636**	−.486*
Intensity R				−.659**	−.631**	.603**
Pleasantness C					.988***	−.881***
Attractiveness C						−.866***
Intensity C						

*Note*. Asterisks indicate level of significance; *p < .05. **p < .01. ***p < .001.

**Table 4.** Correlation Between Ratings of Four-Composite Body Odour Samples (Rated—R) and Individual Odours (Calculated—C).

	Pleasantness R	Attractiveness R	Intensity R	Pleasantness C	Attractiveness C	Intensity C
Pleasantness R		.981***	−.936***	.783*	.805**	−.638
Attractiveness R			−.913**	.864**	.873**	−.741*
Intensity R				−.672*	−.703*	.589
Pleasantness C					.992***	−.914**
Attractiveness C						−.928***
Intensity C						

*Note*. Asterisks indicate level of significance; *p < .05. **p < .01. ***p < .001.

### Correlation Between Ratings of Characteristics

Very strong positive correlations between pleasantness and attractiveness ratings (two-samples: *r* = .998, *p* < .001; four-samples: *r* = .981, *p* < .001), and very strong negative correlations between pleasantness and intensity (two-samples: *r* = −.862, *p* < .001; four-samples: *r* = −.936, *p* < .001) and between attractiveness and intensity (two-samples: *r* = −.856, *p* < .001; four-samples: *r* = −.913, *p* = .001) were found (for more details, see Tables 3 and 4).

## Discussion

The main aim of this study was to test whether composite body odours are rated more positively as compared with the individual samples collected from the same individuals. The rationale was that composite body odours might be more positively perceived because they would be more “average,” as analogously observed in facial attractiveness studies, and perhaps because they perceptually mimic the odour of relatively heterozygous individuals. Contrary to expectation, we did not find significant differences between ratings of the individual samples and both two- or four-composite body odours, and the same pattern was observed for ratings of opposite-sex samples. Similarly, we did not find any positive association between the number of individual body odours constituting composite odours and its attractiveness ratings, as has been observed in facial images. However, we did find significant positive correlations between ratings of two- and four-composite body odours and calculated values of individual odours.

Moreover, our results suggest that two-composite body odour samples retain qualities of the constituent individual odours as we found significant differences between the lower and upper halves of the distribution. The differences were not significant for four-composite body odour samples, perhaps as a consequence of limited sample size, but visual inspection of the data (see Figure 3) indicates that four-composite body odour samples do appear to retain the qualities of the individual constituent samples.

As described earlier, our research was inspired by studies on facial attractiveness. These studies systematically show that composite facial images are perceived as more attractive than the constituent images (Langlois & Roggman, 1990). This was also observed to be independent of their higher bilateral symmetry (Rhodes et al., 1999), another factor contributing to perceived attractiveness (Little & Jones, 2003). The attractiveness of composite images thus appears to be due to their higher prototypicality (Rhodes & Tremewan, 1996); that is, they represent an average facial morphology and may thus mimic heterozygosity in individual faces. These processes are, however, far from how the olfactory system perceives chemical mixtures. Several previous studies have shown that odour mixtures might be perceived quite differently from their constituents (e.g., Thomas-Danguin et al., 2014). Some scholars thus refer to odour mixtures as having emergent perceptual qualities that are frequently difficult to predict from the qualities of the constituents. Furthermore, it has been shown that humans, including professional “noses,” perform rather poorly in identifying individual chemicals from odour mixtures (Jinks & Laing, 1999).

Another possible mechanism is that higher attractiveness of facial composites, due to their prototypicality, is frequently perceived as more familiar. Unfortunately, we did not collect these ratings. Thus, whether composite body odours are perceived as more familiar remains an open question.

### Limitations

In our study, we employed as body odour donors and raters women using hormonal contraception to avoid possible fluctuations in body odour attractiveness (Havlíček, Dvořáková, Bartoš, & Flegr, 2006; Kuukasjarvi et al., 2004) and olfactory abilities (Martinec Nováková et al., 2014) during regular menstrual cycling. A previous study has revealed shifts in MHC-related body odour in contraceptives users (Roberts, Gosling, Carter, & Petrie, 2008), and one may thus argue that our null findings might be thus attributable to the fact that we employed contraceptive users. However, a recent meta-analysis did not show significant differences related to preferences for MHC dissimilarity between contraceptive users and nonusers (Winternitz et al., 2017). In any case, the main advantage in employing hormonal contraceptive users as body odour donors was that we aimed to limit the known effect of cyclic fluctuations, which could drastically interfere with the process of creating blends across individual donors of different cycle phases. We suggest that this step should only increase, and not interfere with, the chance to observe a positive effect of composite body odours because it reduces noise in the collected samples. It is also worth noting that studies of facial composite images, which find robust effects, did not similarly control for effects of hormonal contraception in women either contributing facial photographs or ratings (Langlois & Roggman, 1990; Rhodes & Tremewan, 1996; Rhodes et al., 1999); this seems to us to suggest a likely sensory specificity in how composites of odours and faces are perceived.

Another potential limitation is that, when assessing individual odours and two-composite body odour samples, raters were presented with either one complete cotton pad or two halves, but four-composite samples were created using four halved pads. The difference in the amount of presented material may potentially affect perception of body odour intensity. However, this does not seem to be the case, as we did not find any significant differences between pleasantness, attractiveness and intensity ratings of the individual samples and two- and four-odour composites.

### Implications for Future Studies

Several previous studies have employed composite body odours from individuals sharing characteristics of interest, for example, their sexual orientation (Martins et al., 2005) or affective state (Pause et al., 2004), with the unstated assumption that shared features of the individual odours would be perceivable in the composite stimuli. Our results indicate that individual hedonic qualities, at least, are retained in the odour composites, although further study is needed to investigate the retention of other trait-specific cues. We created composites from individual odours similar in their attractiveness, as this was the most efficient way to test the effect in question. However, it remains an open question as to whether composites made of individual odours that are more variable in hedonic quality would be perceived simply as an average of the constituents or whether such mixtures would show some different, emergent quality. Previous studies investigating interactions between individual body odour and perfume have shown that qualities of the resulting blends cannot be reliably predicted (Sobotková et al., 2017). In a related study, one of the components was kept constant (i.e., the odour donors applied the same perfume), nevertheless the variability in hedonic quality of the blends was comparable with that seen in the body odours alone (Lenochová et al., 2012). Whether patterns observed in studies on perfume-body odour blends can be generalized to the blends of individual body odours should be addressed in future studies.

Remarkably, it has been repeatedly shown that even though perceived qualities of body odour samples tend not to differ during hedonic assessment (e.g., pleasantness), they do induce other effects on individuals exposed to these stimuli, for example, in their autonomic response (Adolph, Schlösser, Hawighorst, & Pause, 2010), anxiety levels (Albrecht et al., 2011) or cognitive functioning (Chen, Katdare, & Lucas, 2006; Gelstein et al., 2011). This evidence suggests that lack of consciously perceived differences does not exclude the possibility of odours transferring other socially relevant cues.

Finally, our findings cannot be interpreted as evidence against a link between heterozygosity and attractiveness. Previous studies indicate that individual body odour samples provide cues to heterozygosity (Thornhill et al., 2003), and this might be specifically linked to heterozygosity in MHC genes that is important in MHC-based mate choice providing direct fitness benefits (Havlíček & Roberts, 2009; Winternitz et al., 2017). Nevertheless, our results demonstrate that the validity of composite body odour samples to test hypotheses about heterozygosity might be limited. It also provides a caution for scholars primarily interested in testing evolutionary hypotheses that the proximate mechanisms that underlie interpreted functional outcomes must be treated with extreme care.
